# Characteristics of COVID-19 Patients With SARS-CoV-2 Positivity in Feces

**DOI:** 10.3389/fcimb.2022.853212

**Published:** 2022-04-12

**Authors:** Wenrui Wu, Ding Shi, Xueling Zhu, Jiaojiao Xie, Xinyi Xu, Yanfei Chen, Jingjing Wu, Lanjuan Li

**Affiliations:** ^1^ State Key Laboratory for Diagnosis and Treatment of Infectious Diseases, The First Affiliated Hospital, College of Medicine, Zhejiang University, Hangzhou, China; ^2^ National Clinical Research Center for Infectious Diseases, The First Affiliated Hospital, College of Medicine, Zhejiang University, Hangzhou, China; ^3^ Collaborative Innovation Center for Diagnosis and Treatment of Infectious Diseases, The First Affiliated Hospital, College of Medicine, Zhejiang University, Hangzhou, China; ^4^ Jinan Microecological Biomedicine Shandong Laboratory, Jinan, China

**Keywords:** SARS-CoV-2, fecal-oral transmission, lymphocyte, viral shedding, COVID - 19

## Abstract

**Background:**

SARS-CoV-2 is highly contagious and poses a great threat to epidemic control and prevention. The possibility of fecal-oral transmission has attracted increasing concern. However, viral shedding in feces has not been completely investigated.

**Methods:**

This study retrospectively reviewed 97 confirmed coronavirus disease 2019 (COVID-19) patients hospitalized at the First Affiliated Hospital, School of Medicine, Zhejiang University, from January 19 to February 17, 2020. SARS-CoV-2 RNA in samples of sputum, nasopharyngeal or throat swabs, bronchoalveolar lavage and feces was detected by real-time reverse transcription polymerase chain reaction (RT–PCR). Clinical characteristics and parameters were compared between groups to determine whether fecal RNA was positive.

**Results:**

Thirty-four (35.1%) of the patients showed detectable SARS-CoV-2 RNA in feces, and 63 (64.9%) had negative detection results. The median time of viral shedding in feces was approximately 25 days, with the maximum time reaching 33 days. Prolonged fecal-shedding patients showed longer hospital stays. Those patients for whom fecal viral positivity persisted longer than 3 weeks also had lower plasma B-cell counts than those patients in the non-prolonged group [70.5 (47.3-121.5) per μL vs. 186.5 (129.3-376.0) per μL, *P* = 0.023]. Correlation analysis found that the duration of fecal shedding was positively related to the duration of respiratory viral shedding (R = 0.70, *P* < 0.001) and negatively related to peripheral B-cell counts (R = -0.44, *P* < 0.05).

**Conclusions:**

COVID-19 patients who shed SARS-CoV-2 RNA in feces presented similar clinical characteristics and outcomes as those who did not shed SARS-CoV-2 RNA in feces. The prolonged presence of SARS-CoV-2 nucleic acids in feces was highly correlated with the prolonged shedding of SARS-CoV-2 RNA in the respiratory tract and with lower plasma B-cell counts.

## Introduction

Severe acute respiratory syndrome coronavirus 2 (SARS-CoV- 2) is a novel coronavirus that has now led to a global pandemic and has resulted in huge burdens to world health and economies since December 2019 (Zhu et al., 2020; World Health Organization). The illness caused by the virus is characterized as an acute and infectious respiratory syndrome, which the World Health Organization (WHO) has named coronavirus disease-2019 (COVID-19). Human-to-human transmission of SARS-CoV-2 is mainly dependent on short-range respiratory droplets or aerosols inhaled *via* the respiratory tract (Prather et al., 2020). It was estimated that the global reproductive number (R0) of SARS-CoV-2 approached 4.08 (Yu et al., 2021). While some infections result in atypical pneumonia, the majority of infections are likely asymptomatic or mild (Huang et al., 2020; Poletti et al., 2021). However, asymptomatic individuals potentially carry SARS-CoV-2 and are considered non-negligible carriers of the virus (Chen et al., 2020). This high contagiousness of SARS-CoV-2 poses a great threat to disease control and prevention.

The angiotensin-converting enzyme 2 (ACE2) receptor has been identified as the primary target for SARS-CoV-2 to invade the host (Hoffmann et al., 2020). There are several parts of the human body that express ACE-2 receptors such as lungs (type I and type II alveolar epithelial cells), cardiovascular system, testis, nasal, oral mucosa, nasopharynx (basal layer of the non-keratinizing squamous epithelium), and smooth muscle cells. And enterocytes are also a possible carrier for ACE-2 receptors. This renders the virus able to reproduce in the gastrointestinal (GI) tract. COVID-19 patients can present with common GI symptoms such as nausea, vomiting, and diarrhea, which can also be the initial symptoms of COVID-19 after illness onset (Song et al., 2020; Lin et al., 2020b). Researchers have detected positive nucleic acid tests among COVID-19 patients with or without GI symptoms (Lin et al., 2020a; Cheung et al., 2020). In addition, Xiao et al. successfully isolated live SARS-CoV-2 from the feces of COVID-19 patients, providing evidence of gastrointestinal infection and fecal excretion of the virus (Xiao et al., 2020). Several researchers have reported prolonged viral shedding in feces (Chen et al., 2020; Zhang et al., 2021). Xing et al. found that SARS-CoV-2 RNA can be present in the stools of pediatric patients 8 to 20 days after viral clearance from the respiratory tract (Xing et al., 2020). Lin et al. reported that detectable SARS-CoV-2 nucleic acid in anal swabs was independently related to ICU admission (Lin et al., 2021). Zeng et al. stated that abdominal pain is associated with increased risk of severe COVID-19 based on a meta-analysis of 21 studies (Zeng et al., 2022). Knowledge about SARS-CoV-2 and the disease is accumulating, but data on viral shedding in feces have not been completely investigated. Here, we describe the detailed features of COVID-19 with positive RT–PCR results in feces as well as the clinical significance of persistent fecal shedding.

## Patients and Methods

### Patients

According to the interim WHO guidance (World Health Organization, 2020), confirmed COVID-19 patients hospitalized in the First Affiliated Hospital, School of Medicine, Zhejiang University, from January 19 to February 17, 2020, were retrospectively recruited for this study by the census method. Detection was performed by real-time reverse transcription polymerase chain reaction (RT–PCR) with a SARS-CoV-2 nucleic acid detection kit (Shanghai Biogerm Medical Technology Co. Ltd.) following the manufacturer’s instructions. Patients’ specimens from respiratory tract (either sputum, nasopharyngeal, throat swabs, or bronchoalveolar lavage, according to the sample availability) and feces were daily collected and tested after their hospitalization for SARS-CoV-2 nucleic acid detection. This study was approved by the Institutional Review Board of the First Affiliated Hospital, School of Medicine, Zhejiang University (Number: IIT20200040A).

### Data Collection

The virologic shedding time in respiratory tract or feces was defined as the duration from the time of illness onset to viral shedding cessation. Illness onset was defined as self-reported symptoms that included but were not limited to fever, cough, fatigue, laryngalgia, runny nose, nausea, vomiting, diarrhea, and abdominal pain. For asymptomatic patients, illness onset was defined by the first positive RT-PCR result in respiratory tract. Viral shedding cessation was considered to be the occurrence of two consecutive negative results over a 24-hour period interval.

Epidemiological data (including age, sex, BMI and current smoking status), underlying comorbid conditions, symptoms, laboratory examination at admission, disease progression, and therapeutic regimens were obtained from patient medical records. Acute Physiology and Chronic Health Evaluation II (APACHE II) scores were assessed on admission. All the data were independently reviewed by two researchers to double check the accuracy of data collection.

### Statistical Analysis

Categorical variables are expressed as counts and percentages and were further analyzed using the χ2 test or Fisher’s exact test. Continuous variables are expressed as the mean and standard deviation or median and interquartile range (IQR) depending on the data distribution. In addition, continuous variables with normal distribution were analyzed with Student’s t test, otherwise Mann–Whitney test (Wilcoxon rank-sum test) was employed. Correlation analysis between the fecal shedding time and other variables was assessed using Pearson or Spearman correlation coefficients. A two-tailed P value of <0.05 was considered significant. All analyses were performed with SPSS software (IBM, Armonk, NY).

## Results

### Clinical Characteristics of Patients With SARS-CoV-2 RNA Positivity in Feces

From January 19 to February 17, 2020, a total of 99 laboratory-confirmed patients with COVID-19 were hospitalized in our center. After excluding two patients who were not tested for fecal nucleic acid, 97 eligible patients were enrolled in this retrospective study. Thirty-four (35.1%) of the patients showed detectable SARS-CoV-2 RNA in feces, while 63 (64.9%) patients in the cohort had negative detection results throughout the observation period (**Table 1**). There was no significant disparity in the age distribution between the fecal-negative group (51.25 ± 13.76 y) and the fecal virus-positive group (54.79 ± 16.98 y). Neither sex showed any tendency for fecal virus shedding, as the male percentages were 55.6% and 70.6% in the two groups, respectively. Both groups of patients, with or without detectable SARS-CoV-2 RNA in feces, had similar BMIs. Meanwhile, neither current smoking status nor underlying morbidity (hypertension and diabetes) were comparable between the two groups. A total of 20.6% of patients (7/34) in the fecal virus-positive group complained of digestive symptoms, including diarrhea, nausea, abdominal pain, and vomiting, but only 15.9% of patients (10/63) in the fecal-negative group complained of digestive symptoms.

**Table 1:** Clinical Characteristics of 97 Hospitalized Patients With SARS-CoV-2 Infection.

Characteristic	Total (N=97)	Virus negative (N=63)	Virus positive (N=34)	P Value
**Demographics**				
Age, mean SD	52.49 ± 14.97	51.25 ± 13.76	54.79 ± 16.98	0.269
Sex, male (%)	59 (60.1)	35 (55.6)	24 (70.6)	0.144
BMI, kg/m^2^	24.38 ± 3.51	24.48 ± 3.19	24.19 ± 4.08	0.708
Current smoking	14 (14.4)	6 (9.5)	8 (23.5)	0.116
Hypertension	33 (34.0)	20 (31.7)	13 (38.2)	0.520
Diabetes	16 (16.5)	10 (15.9)	6 (17.6)	0.822
**Digestive symptoms**	17 (17.5)	10 (15.9)	7 (20.6)	0.560
**Initial laboratory findings**				
PaO2/FiO2, mmHg (IQR)	269.4 (167.4-374.7)	277.2 (167.5-377.5)	254.7 (163.9-369.6)	0.376
Leukocyte count,10^9^/L	5.7 (4.0-9.3)	5.1 (3.4-7.9)	7.9 (5.2-11.2)	0.000
Lymphocyte count, 10^9^/L	0.8 (0.5-1.2)	0.9 (0.6-1.3)	0.6 (0.4-0.9)	0.002
Neutrophil count, 10^9^/L	4.1 (2.6-8.0)	3.4 (2.2-6.6)	6.8 (3.8-10.5)	0.000
Hemoglobin, g/L	134.34 ± 17.60	132.57 ± 17.17	137.62 ± 18.16	0.179
Platelet count, 10^9^/L	188.0 (156.0-241.0)	186 (160-252)	193 (151-236)	0.979
CHOL	3.74 ± 0.77	3.58 ± 0.71	4.05 ± 0.79	0.004
HDL	1.02 (0.84-1.23)	0.97 (0.80-1.16)	1.11 (0.95-1.27)	0.009
TG	1.20 (0.90-1.70)	1.10 (0.86-1.60)	1.40 (1.12-1.76)	0.051
ALT, U/L	21.0 (15.0-31.5)	21 (14-30)	21 (15.8-40.5)	0.591
ALB, g/L	38.69 ± 5.77	38.95 ± 5.68	38.19 ± 5.97	0.538
Cr, umol/L	74.0 (61.0-88.5)	73 (59-88)	77.5 (64-90)	0.276
CK, U/L	69.0 (48.5-111.5)	69 (50-111)	67.5 (43.5-120.5)	0.631
LDH, U/L	247.0 (206.5-336.5)	247 (211-342)	254.5 (202.0-326.5)	0.985
PCT, ng/mL	0.05 (0.03-0.09)	0.06 (0.03-0.08)	0.05 (0.03-0.09)	0.579
CRP, mg/L	18.18 (8.19-48.64)	18.1 (7.6-46.4)	23.5 (8.9-50.3)	0.568

IQR, interquartile range; BMI, body mass index; CHOL,cholesterol; HDL, high density lipoprotein; ALT, alanine aminotransferase; ALB, albumin; Cr, creatinine; AST, aspartate aminotransferase; LDH, lactate dehydrogenase; CRP, C-reactive protein; PCT, procalcitonin; CK, creatine kinase; IgG, immunoglobulin G; NK cell, natural killer cell.

### Laboratory Findings of Patients With SARS-CoV-2 RNA Positivity in Feces

The laboratory results on admission were compared between patients who shed SARS-CoV-2 RNA in feces and those who did not. Blood leukocyte counts in the fecal-positive group were higher than those in the fecal-negative group, which was mainly attributed to the higher neutrophil counts [fecal positive vs. negative, 6.8 (3.8-10.5) vs. 3.4 (2.2-6.6), P < 0.000]. However, the lymphocyte count was significantly depleted in patients who shed fecal virus compared to the lymphocyte count of those without virus RNA detected in their feces [fecal positive vs. negative, 0.6 (0.4-0.9) vs. 0.9 (0.6-1.3), P < 0.05]. Surprisingly, serum concentrations of both cholesterol (CHOL, P < 0.01) and high-density lipoprotein (HDL, P < 0.01) were markedly elevated in the fecal-positive group, and their triglycerides (TGs) also showed a rising trend (P = 0.051). There were no apparent differences in alanine aminotransferase (ALT), creatinine, or creatine kinase between the two groups. Systemic levels of procalcitonin (PCT) and C-reactive protein (CRP) were slightly increased in both groups, with no significant difference.

### Treatment and Outcomes of Patients With SARS-CoV-2 RNA Positivity in Feces

The median duration of fecal viral shedding was approximately 25 days, with a maximum duration of 33 days (**Table 2**). We then measured the duration of these same patients’ viral shedding from the respiratory tract, and we observed that patients with detectable SARS-CoV-2 in feces also had conspicuously longer durations (median time 22 days) of viral shedding from the respiratory tract than the durations of respiratory viral shedding among patients from the fecal-negative group (median time 16 days). Corticosteroid usage was 76.2% in the fecal-negative group and 85.3% in the fecal-positive group (*P* = 0.290). There was no meaningful variation between the two groups in the length of time from illness onset to the start of corticosteroid treatment. Our antiviral regimens mainly included arbidol (200 mg 3 times daily) and lopinavir and ritonavir (LPV/RTV, 400 mg twice daily and 100 mg twice daily, respectively). Darunavir (800 mg once daily) was prescribed if patients suffered apparent side effects with LPV/RTV. Most patients (84.5%) were prescribed antiviral drugs with a combination of two ARV (Antiretroviral) regimens, which was comparable between the two groups (84.1% in the fecal-negative group, 85.3% in the fecal-positive group). Antibiotics and intravenous immunoglobulin treatments made no difference in fecal SARS-CoV-2 shedding.

**Table 2:** Treatment and Outcome of 99 Hospitalized Patients With SARS-CoV-2 Infection.

Variable	Total (N=98)	Virus negative (N=63)	Virus positive (N=34)	P Value
**Virological shedding, d**				
Time of respiratory viral shedding	17 (13-25)	16 (13-21)	22 (16.75-31.25)	0.001
Time of fecal viral persistent positive	/	/	25 (18-33)	/
**Corticosteroid usage**	77 (79.4)	48 (76.2)	29 (85.3)	0.290
Days from illness onset to corticosteroid start	8 (6-10)	8 (7-10)	7 (4.0-9.5)	0.161
**ARV usage**				
Two ARV combination therapy	82 (84.5)	53 (84.1)	29 (85.3)	0.879
**Antibiotic usage**	46 (47.4)	28 (44.4)	18 (52.9)	0.424
Time from illness onset to antibiotic start, d	8 (3.75-11.0)	7.5 (4.0-10.75)	9 (2-11.25)	0.979
**Intravenous immunoglobulin**	39 (40.2)	24 (38.1)	15 (44.1)	0.564
Time from illness onset to IVIG start, d	8 (6.0-9.25)	8 (7-10.5)	7.5 (5.5-9.25)	0.614
Days from illness onset to admission	7 (4-10)	7 (4-10)	7 (4-10.25)	0.659
Length of hospital stay, d	16.60 ± 6.10	15.34 ± 5.74	19.71 ± 5.93	0.003
APACHE II at admission	6 (3-11)	5 (3-11)	7 (3-11.25)	0.151
Admission to intensive care	25 (25.8)	14 (22.2)	11 (32.4)	0.276
Mechanical ventilation	10 (10.3)	6 (9.5)	4 (11.8)	0.737
ECMO usage	7 (7.2)	3 (4.8)	4 (11.8)	0.236

ARV, antiviral treatment; APACHE II, Acute Physiology and Chronic Health Evaluation II; ICU, Intensive Care Unit; ECOMO, extracorporeal membrane oxygenation.

The APACHE II scores assessed on admission showed no apparent difference between patients with or without viral shedding in their feces. The rate of transfer to the ICU, application of mechanical ventilation and use of ECMO were comparable between the two groups.

### Risk Factors for Prolonged Fecal Shedding in COVID-19

**Figure 1:**
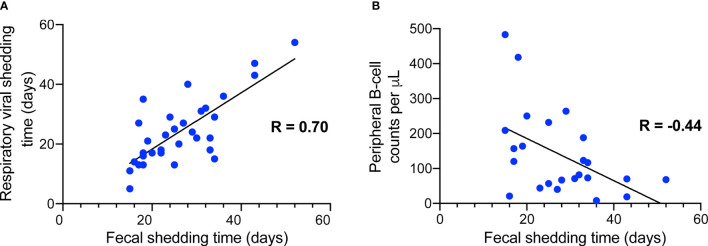
Correlation analysis between fecal shedding duration and respiratory shedding duration **(A)** as well as plasma B-cell counts **(B)**.

It was reported that SARS-CoV-2 virus nucleic acid could persistently test positive in feces after it had been cleared from the respiratory tract. To better characterize the clinical features of prolonged shedding in feces, we divided the 34 patients into two groups according to whether their fecal viral positivity persisted longer than 3 weeks. It was observed that patients with prolonged SARS-CoV-2 shedding in feces started to be prescribed corticosteroids later than the non-prolonged group (8.1 ± 3.8 d vs. 4.7 ± 3.2 d from illness onset, *P* = 0.024, **Table 3**). Additionally, the prolonged fecal-shedding patients showed lower B-cell counts than the non-prolonged group [70.5 (47.3-121.5) per μL vs. 186.5 (129.3-376.0) per μL, *P* = 0.023]. In addition, the prolonged fecal-shedding group also had longer hospital stays and longer respiratory viral shedding times (28.2 ± 10.8 d vs. 17.2 ± 7.8 d, *P* = 0.004). Correlation analysis identified that the fecal shedding duration was positively related to the respiratory viral shedding duration (**Figure 1A**, R = 0.70, *P* < 0.001) and negatively related to peripheral B-cell counts (**Figure 1B**, R = -0.44, *P* < 0.05).

**Table 3:** Factors associated length of fecal SARS-COV2 RNA shedding in the 34 patients.

Characteristics	<21 d (N=12)	>21 d (N=22)	P Value
**Demographics**			
Age	60.5 (32.3-65.8)	58.0 (45.8-67.8)	0.691
Sex, male (%)	7 (58.3)	17 (77.3)	0.247
Current smoking	5 (41.7)	3 (13.6)	0.066
Hypertension	3 (25.0)	10 (45.5)	0.241
Diabetes	3 (25.0)	3 (13.6)	0.406
Corticosteroid usage	10 (83.3)	19 (86.4)	0.812
Days from illness onset to corticosteroid start	4.7 ± 3.2	8.1 ± 3.8	0.024
Antibiotic usage	6 (50.0)	12 (54.5)	0.800
IVIG	5 (41.7)	10 (45.5)	0.832
Time of respiratory viral shedding	17.2 ± 7.8	28.2 ± 10.8	0.004
Time of fecal viral shedding	17.4 ± 1.5	31.3 ± 7.6	<0.001
Length of hospital stay	16.3 ± 4.8	22.6 ± 5.3	0.006
Apach II	6.0 (2.0-11.0)	8.0 (4.5-11.3)	0.294
**laboratory findings on admission**			
PaO2/FiO2, mmHg	270.3 ± 125.2	259.4 ± 130.0	0.815
Leukocyte count,10^9^/L	8.6 (7.2-10.4)	7.7 (5.0-13.6)	0.857
Neutrophil count, 10^9^/L	7.3 (5.6-9.5)	6.7 (3.6-12.7)	0.914
Hemoglobin, g/L	139.2 ± 16.0	136.8 ± 19.5	0.719
Platelet count, 10^9^/L	194.0 (116.3-231.8)	192.0 (155.3-247.3)	0.576
Lymphocyte count, 10^9^/L	0.60 (0.40-0.88)	0.60 (0.40-0.93)	0.942
CD3+ T cell,/μL	324.5 (255.8- 1136.0)	240.0 (111.5-341.8)	0.076
CD4+ T cell,/μL	146.5 (124.8-494.8)	89.0 (52.8-192.3)	0.111
CD8+T cell,/μL	148.0 (120.0-440.0)	130.5 (44.3-154.8)	0.142
B cell,/μL	186.5 (129.3-376.0)	70.5 (47.3-121.5)	0.023
NK cell,/μL	135.9 ± 99.5	111.7 ± 64.1	0.477
Cr, umol/L	67.0 (53.5-83.8)	82.0 (69.3-96.8)	0.044
ALT, U/L	23.0 (15.5-42.3)	20.5 (15.8-40.5)	0.665
CHOL	4.19 (3.92-4.60)	3.78 (3.50-4.29)	0.121
HDL	1.14 ± 0.26	1.12 ± 0.28	0.153
TG	1.66 ± 0.86	1.42 ± 0.58	0.344
LDH, U/L	258.8 ± 65.5	279.2 ± 95.0	0.513
CRP, mg/L	13.9 (9.4-34.6)	37.2 (8.2-66.0)	0.149
PCT, ng/mL	0.04 (0.02-0.09)	0.05 (0.03-0.14)	0.456

## Discussion

It is now believed that respiratory droplets and fomites are the primary transmission routes for SARS-CoV-2. Current disease surveillance mainly focuses on the negative conversion of SARS-CoV-2 RNA in respiratory specimens, as well as the improvement in symptoms, laboratory test results, and radiological abnormalities. However, recovered patients showed detectable SARS-CoV-2 RNA in feces regardless of their symptoms. The digestive tract might be an important organ to excreting the virus (Galanopoulos et al., 2021; Zhou et al., 2022). The possibility of fecal-oral transmission remains to be revealed. This retrospective study described the characteristics of patients whose feces tested positive for SARS-CoV-2 RNA. It was observed that COVID-19 patients could shed SARS-CoV-2 in feces and that these patients presented similar clinical characteristics and outcomes as those who tested negative for SARS-CoV-2 RNA in their feces. The duration of detectable nucleic acid in feces was highly associated with prolonged shedding of SARS-CoV-2 in the respiratory tract and with lower B cells.

Xiao et al. reported that 53.4% of patients with COVID-19 had positive stool RNA results, whereas some of them even had virus-negative respiratory specimens (Xiao et al., 2020). This was consistent with our findings, and prolonged fecal virus shedding might be a noteworthy source of transmission even after the respiratory virus has been cleared. The excretion of SARS-CoV-2 in stools might increase the risk of environmental contamination and facilitate viral spread among the general population through fecal-oral transmission (Arslan et al., 2020; Zhou et al., 2022). The concentration of SARS-CoV-2 RNA in feces was estimated to be 10^4^-10^8^ per gram (Bar-On et al., 2020). It was demonstrated that SARS-CoV-2 can replicate in intestinal organoids and enterocytes to produce new infectious viral particles (Lamers et al., 2020). Researchers found that the gut microbiota of patients with high fecal SARS-CoV-2 activity harbored more opportunistic pathogens (Zuo et al., 2021) and that intestinal barrier function could also be compromised due to disrupted tight junctions (Guo et al., 2021). Gut microbiota were found to be related to the occurrence of complications in COVID-19 patients and might influenced the disease severity (Schult et al., 2022). In addition, not all patients with stools testing positive for SARS-CoV-2 nucleic acid presented symptoms of gastrointestinal infection with SARS-CoV-2 (Han et al., 2020). Tao et al. observed that 47% of patients with COVID-19 showed active infection of SARS-CoV-2 in the gastrointestinal tract even without manifesting GI symptoms (Zuo et al., 2021). In our cohort, among the 34 fecal-positive patients, only 20.6% complained of gastrointestinal symptoms. More research should be conducted to reveal the relationships underlying these observations.

Previous studies have shown that corticosteroid treatment could prolong the viral shedding time of SARS, MERS, H7N9, and even severe influenza (Lee et al., 2004; Lee et al., 2009; Arabi et al., 2018; Wang et al., 2018). Similarly, researchers found that the proportion of patients prescribed corticosteroids was lower among the patients with early SARS-CoV-2 RNA clearance than among the patients with late virus RNA clearance (Xu et al., 2020). This might be associated with the immune-suppressing effects of corticosteroid therapy (Arabi et al., 2018). In our research, we found that the earlier patients used corticosteroids (from the time of illness onset), the shorter was the duration of fecal viral shedding. We believe that this was related to the lower doses of corticosteroids (initial dosage in fecal virus-positive patients, 61.29 ± 23.63 mg/d methylprednisolone) administered to patients in our research group, as it was demonstrated in an earlier report that low-dose corticosteroid therapy did not delay viral clearance in patients with COVID-19 (Fang et al., 2020). The latest research has shown that the administration of systemic corticosteroids can reduce 28-day all-cause mortality (WHO Rapid Evidence Appraisal for COVID-19 Therapies (REACT) Working Group et al., 2020), which indicates the therapeutic role of corticosteroids in COVID-19 treatment. More research needs to be done to determine the optimal dosage and duration of treatment with corticosteroids.

Our study found decreased lymphocyte and increased neutrophil counts in the fecal-positive group. It was reported that increased neutrophil counts were predominantly found in critically ill patients and were related to poor prognosis (Chen et al., 2020). La et al. observed that the neutrophil/lymphocyte ratio could be a prognostic factor for COVID-19 with a high ratio suggesting worse survival (La Torre et al., 2022). Elevated peripheral neutrophil counts are associated with cytokine storms and massive neutrophil infiltration of the lungs, resulting in severe lung lesions (Channappanavar and Perlman, 2017). A recent study revealed the distinct characteristics of the viral coding proteins of SARS-CoV-2, and infection with the virus could induce enrichment of host responsive genes related to the overactivation of neutrophils (Qin et al., 2021). This finding suggested the crucial roles of neutrophil dysfunction in COVID-19, which was also found to be a distinct inflammatory feature related to the pathogenesis of SARS and MERS (Channappanavar and Perlman, 2017). However, our study did not observe disparities in disease severity between the fecal shedding group and the other group, which might be due to the limited number of enrolled patients and the fact that only the APACHE II score on admission was analyzed. B lymphocytes and CD4+ and CD8+ T lymphocytes were reported to be reduced in severely ill and critically ill COVID-19 patients, which could be related to patient outcomes (Sun et al., 2020; Huang et al., 2020). Seroconversion for IgG occurred in approximately 50% of patients by day 7 after disease onset, suggesting that B-cell immunity was involved in SARS-CoV-2 infection (Wolfel et al., 2020). Plasma cells were significantly increased in recovering COVID-19 patients, while naïve B cells were significantly decreased (Wen et al., 2020). Lower B-cell counts might lead to poor virus elimination and severe disease (Kotagiri et al., 2022). More research could be conducted to examine the long-term dynamics of B cells and SARS-CoV-2-specific antibodies produced *in vivo*.

In our correlation analysis, we found a positive correlation between respiratory and digestive tract viral shedding. Continuous positive virus detection in respiratory samples was related to disease severity (Shi et al., 2020) and led to longer hospital stays, and our study found that patients with fecal positive RNA results or those with a persistent and longer fecal shedding duration had longer hospital stays. Although it is still uncertain how SARS-CoV-2 infects extrapulmonary tissue, whether *via* the circulatory or lymphatic pathways, scientists have stated that the pathophysiology of COVID-19’s extrapulmonary manifestations might involve endothelial damage and thrombo-inflammation, dysregulated immune reactions, or disturbance of ACE2-related pathways (Gupta et al., 2020). Delayed viral clearance from the respiratory system provides greater opportunity for the virus to invade extrapulmonary tissue, including the digestive tract.

There were several limitations in this study. First, this was a single-center retrospective study, and our sample size, especially that for patients with positive fecal results, was limited, which may cause bias. Second, this study analyzed only laboratory findings on admission, whereas subsequent alterations in the patient condition might influence the process of fecal viral shedding.

In conclusion, this study found that SARS-CoV-2 RNA could be detected in feces, and fecal-positivity was independent from the manifestation of GI symptoms or disease severity. The prolonged shedding of SARS-CoV-2 nucleic acids in feces was related to the prolonged duration of viral shedding in the respiratory tract and to lower plasma B-cell counts. Fecal SARS-CoV-2 positivity should be given more attention due to its potential for environmental contamination, and more research is warranted to further establish a fecal-oral transmission and to reveal the dynamic concentration of viral shedding from the GI tract.

## Data Availability Statement

The original contributions presented in the study are included in the article/supplementary material. Further inquiries can be directed to the corresponding author.

## Ethics Statement

The studies involving human participants were reviewed and approved by Institutional Review Board of the First Affiliated Hospital, School of Medicine, Zhejiang University. Written informed consent for participation was not required for this study in accordance with the national legislation and the institutional requirements.

## Author Contributions

WW and LL participated in the study design. WW and DS performed the statistical analysis and wrote the paper. XZ, JX, XX, and JW recruited the patients and collected clinical data. YC and LL conceived of the study and helped to draft the manuscript. All authors contributed to the article and approved the submitted version.

## Funding

This study was funded by the National Key Research and Development Program of China (2018YFC2000500) and the National Natural Science Foundation of China (82100601, 81790613 and 81570512) and Research Project of Jinan Microecological Biomedicine Shandong Laboratory (JNL-2022001A).

## Conflict of Interest

The authors declare that the research was conducted in the absence of any commercial or financial relationships that could be construed as a potential conflict of interest.

## Publisher’s Note

All claims expressed in this article are solely those of the authors and do not necessarily represent those of their affiliated organizations, or those of the publisher, the editors and the reviewers. Any product that may be evaluated in this article, or claim that may be made by its manufacturer, is not guaranteed or endorsed by the publisher.
